# The role of thallium-201 and pentavalent dimercaptosuccinic acid for staging cartilaginous tumours

**DOI:** 10.1186/1477-7800-1-10

**Published:** 2004-11-08

**Authors:** Peter FM Choong, Toshiyuki Kunisada, John Slavin, Stephen Schlicht, Rodney Hicks

**Affiliations:** 1 Department of Orthopaedics, University of Melbourne, St. Vincent's Hospital, Melbourne, Australia; 2 Department of Medical Imaging, St. Vincent's Hospital, Melbourne, Australia; 3 Department of Pathology, St. Vincent's Hospital, Melbourne, Australia; 4 Department of Medical Imaging, Peter MacCallum Cancer Institute, Melbourne, Australia; 5 Division of Surgical Oncology, Peter MacCallum Cancer Institute, Melbourne, Australia

## Abstract

**Introduction:**

Heterogeneity of cartilage tumours may confound accurate diagnosis and grading resulting in under and over treatment. Improved preoperative assessment of malignancy and grade would be invaluable for developing a rational plan for treatment. We examined correlations between nuclear tracer avidity and malignancy grade in cartilage tumours.

**Methods:**

Between 1996 and 2000, 92 consecutive patients with cartilaginous tumours (50 benign, 42 non-metastatic malignant) underwent nuclear scanning. Thallium-201 (TL-201) and pentavalent dimercaptosuccinic acid (DMSAV) were used as nuclear isotopes. Scanning with these agents was performed on separate days 48 hours apart. Static and SPECT images were obtained at 30 m and 4 h after injection of nuclear tracer. Pathology review was undertaken blinded to the results of the nuclear scans and correlations between histologic results and trace uptake at 4 hours examined.

**Results:**

25 patients with negative DMSAV had benign tumours. 15/17 tumours with positive TL-201 had malignant tumours. 11/13 patients with both positive DMSAV and TL-201 scans had intermediate or high grade tumours and 4 of these developed metastases. We have developed an algorithm for the management of patients with tumours that aims to avoid over treatment of low grade tumours and under treatment of high grade tumours.

**Conclusion:**

Functional nuclear scanning with TL-201 and DMSAV complements other imaging modalities in the management of cartilaginous tumours.

## Background

Traditionally, the determination of malignancy and its grade in cartilage tumours has been from the combination of history, radiographic features with or without computed tomography and histologic examination [1-3]. More recently, magnetic resonance imaging has also been employed [4-6]. However, cartilage tumours are recognized for their histologic heterogeneity [7-9] and bizarre physical features, such that reliance on anatomic imaging and accuracy of biopsy alone for interpretation of state of malignancy or benignity may be misleading. While the recognition of high grade malignancy is not difficult, the differentiation between benign and low grade (grade I) tumours can present a diagnostic dilemma [10,11]. Such a dilemma may lead inadvertently to under-or over-treatment of cartilage tumours.

Functional nuclear scans employ isotopes that become substrates for various cellular metabolic cycles and therefore, are useful for determining the metabolic activity in these tissues [12]. Given that malignant tumours are more metabolically active than benign tumours, and that there is a relationship between grade of malignancy and metabolic activity, functional nuclear scans may help to differentiate between cartilage tumours of varying metabolic activity, which in turn, may shed some light on their state of malignancy.

We now report our experiences with 2 radio-siotopes, Thallium-201 (Tl-201) and pentavalent dimercaptosuccinic acid (DMSAV) for determining the metabolic activity of cartilage tumours, and their value in differentiating between malignant and benign cartilage tumours in 92 consecutive cartilage tumours. We describe their role in the development of our current regimen for treating cartilage tumours.

## Methods

### Patients

Between January 1997 and December 2000, 92 consecutive patients were referred to our institution for investigation and management of suspected chondral tumours. There were 50 females and 42 males with a median age of 45 years (range 16–87 years).

Tumours were located in the humerus (17), chest wall (3), femur (27), hand (3), knee (3), vertebra (1), scapula (7), tibia (16), pelvis (9), foot (4), radius (2). All patients had previously unoperated tumours and no patient presented with metastases.

### Investigations

All patients were examined with plain radiographs, computed tomography and magnetic resonance imaging. From 1997 onward, TL-201 and/or DMSAV scans were also performed on patients with suspected chondral tumours.

Tl-201 and DMSAV scintiigraphy were conducted at two centers (SVH, PMCI) with similar imaging protocols. All studies were conducted using a gamma camera. A pre-determined dose of radioisotope was administered intravenously and scintigraphic images obtained at 30 minutes (early phase) and 3–4 hours (late phase) after injection in all cases. Early phase static images were acquired over the area of interest and late phase images consisted of whole body imaging and SPECT over the area under investigation. A low energy high resolution, parallel-hole collimator was used and image acquired in a 128 × 128 matrix for 5 minutes. A simple grading system was devised for late phase isotope uptake with NO UPTAKE indicating no tumour uptake greater than background activity and INCREASED UPTAKE indicating definite activity greater than the background level. The background level referred to is that of the tissue within which the tumour arose, that is, bone. We selected the late phase results for correlation with the results of histological examination of the surgical specimens because early uptake at 30 minutes may also represent peritumoural inflammation or vascularity, which may confound the interpretation of results. In contrast, late phase uptake represented true tumour uptake of isotope.

### Tumours

The pathologist (JS) is the designated lead pathologist on the Victorian Bone Tumour Registry. For the purpose of this study, assessments of surgical tissue and histologic re-evaluation were conducted without prior knowledge of the results of the functional nuclear scans. There were 50 benign tumours and these consisted of enchondromata (29), osteochondromata (17), chondroblastoma (3), and chondromyxoid fibroma (1). There were 42 malignant tumours and these consisted of 38 central chondrosarcoma and 4 dedifferentiated chondrosarcoma. Of these malignant tumours, 22 were graded as grade I, 12 as grade II and 8 as grade III according to.

### Treatment

All tumours in this study were treated with excision. In those tumours that were considered to be benign or grade 1 malignancy as based on history, examination, plain radiography, magnetic resonance imaging and functional scanning, were treated by careful intralesional curettage, burring with a high speed dental burr, pulsatile lavage, chemical cautery with phenol and then the defect filled with polymethylmethacrylate cement. In those tumours that were considered to be clearly malignant and interpreted as grade 2 and higher or if the functional nuclear scans showed significant uptake, wide resection was employed.

Because of the histologic heterogeneity of cartilage tumours, the majority of tumours were not biopsied prior to definitive surgery. Biopsy was considered if there was doubt about the diagnosis, or if the anticipated treatment was potentially far greater than may have been required. If biopsy was preferred but could not be safely performed preoperatively, for example, a periacetabular tumour, frozen section drill biopsy would be conducted as part of the initial surgical approach to the tumour.

### Follow-up

No patients were lost to follow-up. Patients with malignant tumours were reviewed every 3 months for the first 2 years and 6 monthly after that for a further 2 years with a plan for yearly review for the following 4 years. Computed tomography of the chest was performed every 6 months, and plain radiographs obtained of the operated area at each visit.

The median follow-up was 3.7(0–6) years. At last review, 85 patients were alive without disease, 2 were alive with pulmonary metastases. 3 patients had died of metastatic disease, one patient died from intraoperative complications and 1 patient died from a pulmonary embolus 1 month after surgery. There were no local recurrences.

## Results

### Thallium scans

Eighty seven of ninety two patients underwent thallium scanning. Of these, 17 patients had increased uptake on the delayed scans, and 70 patients had no uptake on the delayed scans.

### DMSA(V) scans

Eighty three of ninety two patients underwent DMSA(V) scanning. Of these, fifty eight patients had increased uptake on the delayed scans and twenty five patients had no uptake on the delayed scans.

### Combined thallium and DMSA(V) scans

Seventy eight of ninety two patients had both thallium and DMSA(V) scanning. There was no uptake with Thallium and DMSA(V) scanning in twenty patients. Thallium scanning was negative and DMSA(V) was positive in forty five patients. There was uptake on both Thallium and DMSA(V) scanning in 13 patients. There was no case where there was thallium uptake without DMSA(V) uptake.

### Correlation between functional scanning and state of benignity /malignancy of tumours

*a. Thallium scans *Of the 50 benign tumours, 45 underwent thallium scanning. Forty-three tumours had negative scans and 2 had positive scans. Of the 42 malignant tumours, 27 had negative thallium scans and 15 had positive scans.

*b. DMSA(V) scans *Of the 50 benign tumours, 47 had DMSA(V) scans. Twenty five of these tumours had negative scans and 22 had positive scans. Of the 42 malignant tumours, 36 had DMSA(V) scans. All 36 of these were positive. No malignant tumour was DMSA(V) negative.

*c. Combined thallium and DMSA(V) scans *There 78 tumours that were scanned with both thallium and DMSA(V). Of the 42 benign tumours, 2 had positivity forboth thallium and DMSA(V), 20 had positivity only for DMSA(V) and 20 had no uptake on either scan.

Of the 36 malignant tumours that were scanned with both isotopes, 11 showed positivity for both thallium and DMSA(V), while 25 tumours showed positivity for DMSA(V) only. No malignant tumour was negative to thallium scanning.

### Correlation between functional scanning and tumour grade in 42 choindrosarcomas

*a. Thallium scans *Of the 42 chondrosarcomas, 7 grade II and 8 grade III tumours had positive thallium uptake. No grade I tumour had thallium uptake. Twenty two grade I tumours, and 5 grade II tumours showed no uptake.

*b. DMSA(V) scans *Thirty six of the 42 chondrosarcomas had DMSA(V) scans. All were positive (20 grade I, 11 grade II, 5 grade III). No chondrosarcoma was DMSA(V) negative.

### Correlation between functional scanning and metastasis

No patient with a negative thallium scan developed metastasis. Six of seventeen patients with positive thallium scans developed metastases. In contrast, four of fifty eight patients with positive DMSA(V) scans developed metastases. In patients where both scans were performed, four of thirteen patients developed metastases.

## Discussion

Anatomic imaging such as computed tomography [13] and magnetic resonance imaging [4-6,14] provide excellent morphologic delineation and localisation of bone tumours. These tests are invaluable in the surgical planning for patients with musculoskeletal tumours However, these tests do not always give an indication of the biologic behaviour of the tumour, particularly if there are subtleties between benign and low grade malignant states [15]. Cartilaginous tumours of bone are characterized by radiologic and histologic heterogeneity [7,8] that may give rise to diagnostic dilemas. Benign tumours such as enchondromas and osteochondromas may appear large and bizarre giving an impression of biologic aggressiveness, particularly those in the hand, while some chondrosarcomas such as a clear cell chondrosarcoma may be small, well defined, slow growing and apart from osteolysis have no other hallmark of malignancy [16-18] such as cortical destruction or soft tissue extension to express its malignant phenotype. One of the major difficulties in orthopaedic oncology is the differentiation between enchondroma and grade 1 chondrosarcoma [19].

Conventional technetium monodiphosphonate skeletal scans are employed to identify uni-or multifocal disease when bone tumours are suspected [12]. For positivity, this scanning modality relies on the interactions between tumour and host bone that incite an osteoblastic response by adjacent bone and does not necessarily imply malignancy. For example, enchondroma and chondrosarcoma frequently demonstrate a similar uptake of nuclear tracer, without differentiating between benignity and malignanc.

Reliance on preoperative histologic diagnosis for determining the nature of a cartilage lesion can also be difficult. Biopsy of a suspicious cartilage tumour is most helpful when clear malignancy is demonstrated because a benign finding does not necessarily exclude malignancy. The histologic heterogeneity of cartilage tumours, however, is well recognized and unless the biopsy accurately targets the most malignant part of the tumour, there is a risk that the histologic diagnosis may under-report the state of malignancy if this should exist.

Functional nuclear scans reflect the metabolic activity of tumours and may provide important information regarding their biologic behaviour. In this regard, hypermetabolic tumour tissue is likely to be more active than surrounding normal tissue and this difference may be valuable for distinguishing between benign and malignant tumours. Similarly, when dealing with grades of malignancy, a higher level of metabolic activity may be expected from high grade tumours in comparison to lower grade counterparts. Since the late 1970s, thallium (Tl-201) scanning has been used extensively as a safe method to assess ventricular function in patients with myocardial ischaemia and the results have been interpreted as reflecting relative levels of myocardial metabolic activity. As Tl-201 is a potassium analogue, its uptake into cells depends on the sodium potassium ATPase dependent pump. Tl-201 is a readily available, cyclotron produced radionuclide that decays by electron capture with a half-life of approximately 73 hours. The liver, spleen, kidneys, myocardium, thyroid, choroids plexus of the lateral ventricles and testis normally demonstrate avidity for Tl-201, with very minimal uptake in healing surgical wounds. Tl-201 is thought to accumulate less well within connective tissue, which contains inflammatory cells and almost undetectable in necrotic tissue. Localization of Tl-201 within tumours appears to be influenced by tumour vascularity, tumour cellularity, the metabolic rate of the tumour and the histological type of tumour. Thallium uptake studies [20-23] in patients with bone tumours have been used to predict response to preoperative chemotherapy by correlating the histological degree of tumour necrosis to changes in Tl-201 uptake. The correlation between thallium uptake and tumour metabolic activity, thus makes it a good candidate tracer to assess malignancy in chondromatous tumours. 99Tcm-Dimercaptosuccinic acid (DMSA) is another readily available isotope, which was first described as an isotopic agent for investigating the renal parenchyma in a variety of disease entities [24-26]. Subsequently, the pentavalent form of 99Tcm-dimercaptosuccinic acid (99Tcm-(V)DMSA) developed a recognised role for imaging medullary thyroid carcinoma, [27-30] and this role was further investigated in relation to assessing bone metastases and other neoplastic conditions such as multiple myeloma, osteosarcoma and chondrosarcoma. [31-33]; [28,34]. Of note, uptake of DMSA(V) is reported to be absent in vertebral collapse and osteoarthritis [35].

Our study has demonstrated that the combined use of Tl-201 and DMSA(V) scanning may be correlated with the benignity, malignancy and grade of cartilaginous tumours. For a tumour that is hetereogeneous not only on radiologic but also pathologic appearance, the use of functional nuclear scanning may help to differentiate particularly between the low grade and benign lesions. Of note, no malignant lesion had a negative DMSA(V) scan, and only 2 out of 45 benign tumours showed thallium uptake in our study.

While this modality of investigation does not replace the value of combining elements from the clinical presentation, plain radiographic changes and pathology, functional nuclear scanning may increase the confidence of diagnosing a low grade or benign tumour, and the availability of information preoperatively may also be helpful for tumours that are deeply situated and difficult to biopsy. With regard to biopsy, localized thallium or DMSA(V) positivity may be useful for guiding tissue sampling from the most metabolically active site, thus enhancing the procurement of potentially the most aggressive/malignant part of the tumour [12].

Although, the median duration of follow up in this study was limited to 3.7 years, the high proportion of metastases in patients whose sarcomas were thallium positive may suggest a role for thallium scanning as a prognostic indicator for metastasis in chondrosarcoma. Interestingly, the combination of DMSA(V) with thallium scanning did not improve the prognostic value for metastases. This may be attributed to the fact that the spread of DMSA(V) positivity amongst the tumours was greater than thallium positivity, whereas the latter was positive only in patients with grade II and III chondrosarcomas, which are the ones that mainly metastasise.

We cannot explain why 2 out of 45 benign tumours were thallium positive. The specimens were reviewed by a panel of pathologists expert in bone pathology and none conferred a diagnosis of sarcoma. In both of these cases, DMSA(V) positivity was also observed. It is important to note that these tumours occurred in the hand and foot, although not part of the syndrome of enchondromatosis. It is appreciated that small bone enchondromata may behave locally in a very aggressive manner without any cytologic evidence of malignancy [19]. However, small bone chondrosarcoma are also know to be fatal and careful inspection of imaging and pathology is required to ensure distinction between benign and malignant lesions which may behave similarly [36,37]. No conclusion can be drawn about the biologic significance of our finding of 2 benign small bone tumours with thallium uptake, in view of the very small numbers of these in our study. One patient had a small toe amputation and the other had curretage, chemical cautery and cementation of a phalangeal lesion. Neither have represented with local or systemic recurrence of disease.

**Figure 1:**
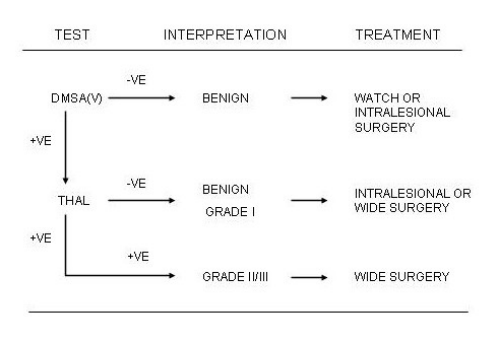
Algorithm for the use of DMSA(V) and thallium scanning for cartilaginous tumours. Thal : Thallium-201; DMSA(V) : Pentavalent dimercaptosuccinic acid +ve : positive uptake; -ve : no uptake.

As a result of this study, we now undertake the following sequence of scanning and treatment (Figure 1). All patients with a suspected chondral tumour would undergo a DMSA(V) scan. If this is negative, no further scanning is performed and the tumour is regarded as benign because our study had indicated that no malignant tumour had a negative DMSA(V) scan. These tumours would then be treated on their individual merits. If there is uptake on the delayed DMSA(V) scans our study had indicated that half of these are likely to be malignant therefore, patients would undergo thallium scanning. If there is no uptake, then based upon our study, the result would be interpreted as benign cartilage tumour or grade 1 chondrosarcoma. Patients may then undergo intralesional curettage with chemical cautery and cementation or wide excision. If there is uptake on the thallium scan, our study demonstrated that there is a very high likelihood that these tumours would be grade II or III and a wide excision would be recommended.

Surgical treatment for cartilage lesions vary widely [38]. Intralesional curettage together with local adjuvant treatment is often recommended for benign tumours but the management of chondrosarcoma follows the same principles as espoused for all sarcomas [39,40]. Wide resection with a cuff of normal tissue radially and axially around the tumour is encouraged. The importance of good margins is highlighted by the predilection for local recurrence of incompletely excised chondrosarcoma [41-43], and also because of the risk of chondrosarcomas recurring at a higher maliginancy grade with its associated increased risk of metastasis [44]. Inaccurate diagnosis and or grading may thus result in under- or over treatment of cartilage neoplasms that may later manifest as troublesome local recurrence or unnecessary loss of function from ablative surgery.

The management of grade 1 tumours remains controversial. Some authors have recommended intralesional curretage, adjuvant treatment and cementation for these lesions [45,46]. In a series of 40 enchondromata and low grade chondrosarcomas, Bauer et al. observed a local recurrence of 0.09 over 10 years using this modality of treatment. In all cases, local control was subsequently achieved by repeating the earlier surgery on the recurrence. Neither recurrence occurred as a higher grade of tumour. In contrast, some have recommended wide excision of grade I lesions and so called "borderline" chondrosarcoma for fear of tumour recurrence at a higher grade, which would portend toward a poorer outcome [47,48]. The relationship between thallium positivity and higher grades of chondrosarcoma in our study suggests that it would be important to review in a prospective manner the outcome of surgery based upon our algorithm of preoperative scanning.

## Conclusion

There has been rising interest in studying non-invasive techniques of imaging cartilage tumours to try and determine their biologic aggressiveness prior to definitive surgery. The advantages of functional nuclear scanning with DMSA(V) and thallium are that they are easy to perform in the majority of nuclear medicine departments, the isotopes are easily available and the costs are not prohibitive. We have found that the information derived from our study complements other imaging modalities and apart from improving our understanding of cartilaginous tumours, has also assisted us in developing a strategy for their treatment.

## Competing interests

The author(s) declare that they have no competing interests.

## List of abbreviations

TL-201 – Thallium -201

DMSA(V) – Pentavalent dimercaptosuccinic acid

## Authors contribution

Prof. Peter Choong Surgeon, carried out surgery, participated in clinical and diagnostic practice. Wrote and prepared manuscript

Dr. Toshikunisada Orthopaedic oncology fellow, collected diagnostic data

Dr. Stephen Schlicht Nuclear physician, Participated in manuscript preparation, and conduct of diagnostic tests

Dr. Rodney Hicks Nuclear physician, Participated in manuscript preparation and conduct of diagnostic tests

Dr. John Slavin Pathologists, participated in blinded review of patients histology
